# The underlying mechanism of proinflammatory NF-κB activation by the mTORC2/Akt/IKKα pathway during skin aging

**DOI:** 10.18632/oncotarget.10943

**Published:** 2016-07-29

**Authors:** Yeon Ja Choi, Kyoung Mi Moon, Ki Wung Chung, Ji Won Jeong, Daeui Park, Dae Hyun Kim, Byung Pal Yu, Hae Young Chung

**Affiliations:** ^1^ Molecular Inflammation Research Center for Aging Intervention, College of Pharmacy, Pusan National University, Busan, Korea; ^2^ Department of Predictive Toxicology, Korea Institute of Toxicology, Daejeon, Korea; ^3^ Human and Environmental Toxicology, School of Engineering, University of Science and Technology, Daejeon, Korea; ^4^ Department of Physiology, The University of Texas Health Science Center at San Antonio, San Antonio, Texas, United States of America

**Keywords:** skin aging, mTORC2, NF-κB, UV, Gerotarget

## Abstract

Mammalian target of rapamycin complex 2 (mTORC2), one of two different enzymatic complexes of mTOR, regulates a diverse set of substrates including Akt. mTOR pathway is one of well-known mediators of aging process, however, its role in skin aging has not been determined. Skin aging can be induced by physical age and ultraviolet (UV) irradiation which are intrinsic and extrinsic factors, respectively. Here, we report increased mTORC2 pathway in intrinsic and photo-induced skin aging, which is implicated in the activation of nuclear factor-κB (NF-κB). UVB-irradiated or aged mice skin revealed that mTORC2 activity and its component, rictor were significantly upregulated which in turn increased Akt activation and Akt-dependent IκB kinase α (IKKα) phosphorylation at Thr23 *in vivo*. We also confirmed that UVB induced the mTORC2/Akt/IKKα signaling pathway with HaCaT human normal keratinocytes. The increased mTORC2 signaling pathway during skin aging were associated to NF-κB activation. Suppression of mTORC2 activity by the treatment of a mTOR small inhibitor or knockdown of RICTOR partially rescued UVB-induced NF-κB activation through the downregulation of Akt/IKKα activity. Our data demonstrated the upregulation of mTORC2 pathway in intrinsic and photo-induced skin aging and its role in IKKα/NF-κB activation. These data not only expanded the functions of mTOR to skin aging but also revealed the therapeutic potential of inhibiting mTORC2 in ameliorating both intrinsic skin aging and photoaging.

## INTRODUCTION

Mammalian target of rapamycin (mTOR) is an evolutionarily conserved nutrient-sensing protein kinase that belongs to the PI3K-related kinase family. The mTOR signaling pathway has attracted considerable attention in the field of aging research because its suppression was found to extend the lifespan of various organisms [1, 2]. However, there is no study to date that examined the function of mTOR signaling pathway in skin aging. Skin aging has a difference with other organs' aging because skin aging is a complex biological process involving both extrinsic factors and intrinsic and chronological aging process. Extrinsically, ultraviolet (UV) irradiation is the most powerful and common environmental factor accelerating skin aging, commonly referred to as photoaging [3].

mTOR forms two different enzymatic complexes, mTOR complexes 1 and 2 (mTORC1 and mTORC2, respectively), that possess distinct functions and components [4]. mTORC2 consists of mTOR, G-protein β-subunit-like protein (GβL, also known as mammalian lethal with sec-13 protein 8), rapamycin-insensitive companion of mTOR (Rictor), stress-activated protein kinase interacting protein 1 (Sin1) and protein observed with Rictor (Protor)-1 or Protor-2. mTORC2 has been identified as the critical upstream kinase responsible for the phosphorylation of Akt at Ser473 and its activation [5]. Therefore, mTORC2 has a great potential to modulate various substrates that are known to be regulated by Akt.

Nuclear factor-κB (NF-κB) is a transcription factor that plays a well-established and important role in photoaging and intrinsic aging. Previously, Akt-mediated activation of NF-κB was reported to occur during T cell activation, tumorigenesis, and the development of invasiveness in cancer cells [6-8]. Akt phosphorylates IκB kinase α (IKKα) at Thr23 inducing its activity which in turn leads to IκB degradation. In addition, Akt-mediated phosphorylation of IKKα at residue Thr23 leads to its translocation to nucleus resulting in the posttranslational modification of p65, an NF-κB subunit, during Hepatitis B virus X-mediated hepatocellular carcinoma progression [9].

The purpose of this study was to delineate the mechanism by which mTORC2-mediated activation of Akt as underlying mechanism of NF-κB activation during skin aging. Our data showed that the mTORC2/Akt/IKKα signaling pathway led to NF-κB activation in both intrinsic and photo-induced skin aging, suggesting that this mechanism can be an ideal target for the prevention of skin aging.

## RESULTS

### Upregulation of mTORC2 activity during skin aging

**Figure 1:**
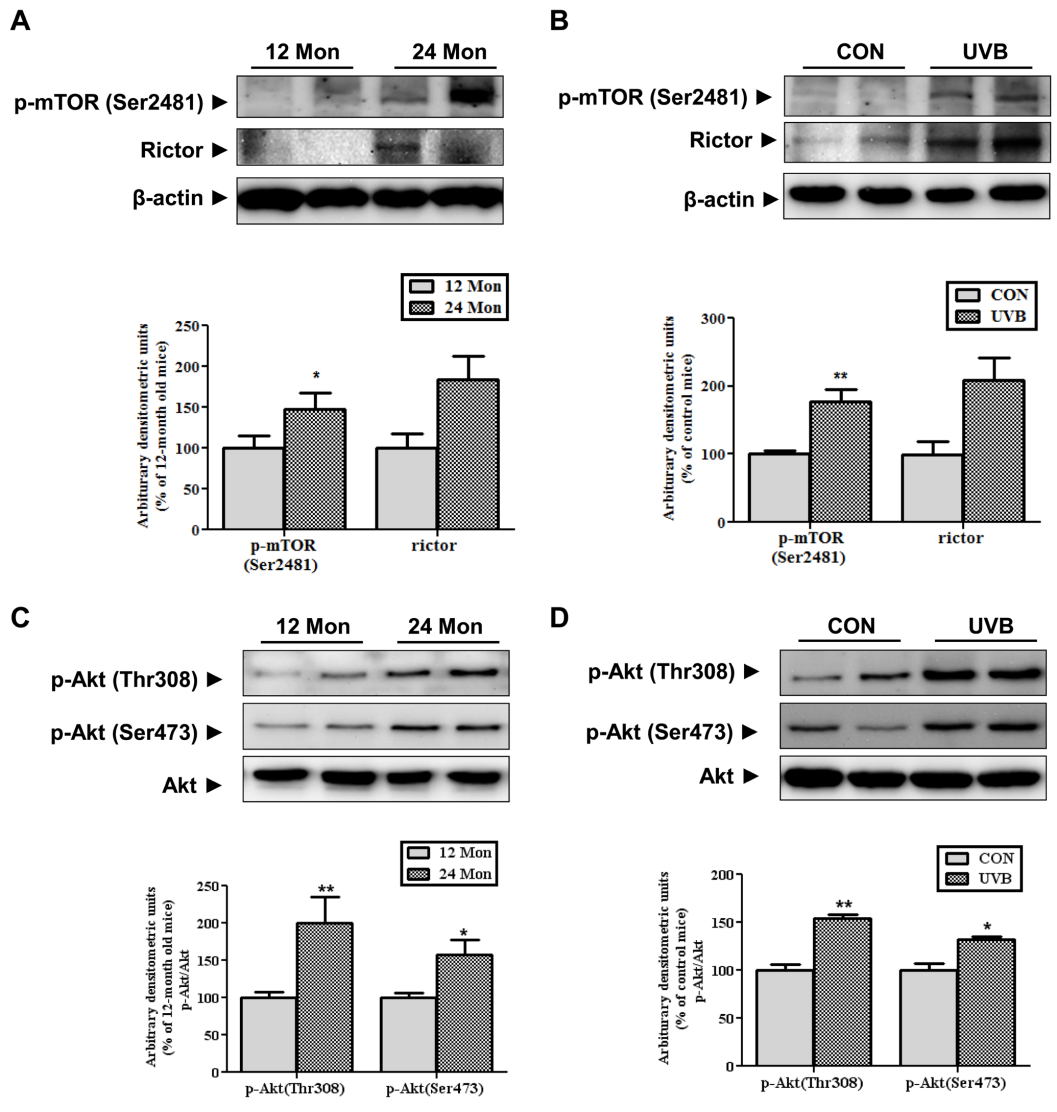
Changes in the expression and activity of mTORC2 in aged skin Western blotting was performed to detect the levels of mTOR phosphorylated at Ser2481, Rictor (**A** and **B**), and activated Akt at Thr308 and Ser473 (**C** and **D**) in skin homogenates of 12- and 24-month-old mice (A and C) and in skin homogenates of control and UVB-irradiated mice (B and D). The blots were quantified by densitometry. The blots of phospho-mTOR (Ser2481) and Rictor were normalized to β-actin and the phosphorylated forms of Akt were normalized to total Akt. Bars represent the mean percentage value ± SEM in 12-month-old mice (*n* = 7-8, * *p* < 0.05, ** *p* < 0.01 *vs*. 12-month-old mice) and control mice (*n* = 7-8, * *p* < 0.05, ** *p* < 0.01 *vs*. control mice).

We verified the mTORC2 activation using both intrinsic and extrinsic skin aging models *in vivo*. We used 12- and 24-month-old C57BL/6 mice and UVB-irradiated Hos:HRM-2 melanin-possessing hairless mice as models of intrinsic skin aging and photoaging, respectively. mTORC1 contains mTOR phosphorylated primarily on Ser2448 whereas mTORC2 contains mTOR phosphorylated primarily on Ser2481 [10], we measured the mTORC2-specific phosphorylation of mTOR and components. The level of phospho-mTOR (Ser2481), which represents mTORC2 activity, and the expression of Rictor, a component of mTORC2, were increased in both the intrinsically aged (Figure 1A) and photo-aged skin (Figure 1B) *in vivo*. mTORC2 directly phosphorylates Akt at Ser 473. Upregulation of Akt phosphorylation was detected in both intrinsically aged (Figure 1C) and photoaged skin (Figure 1D) in accordance with increased mTORC2 activity. These data indicated that mTORC2 activation occurred during not only intrinsic skin aging but also UVB-induced skin aging and suggested an association between mTORC2 and skin aging.

### The levels of phosphorylated of IKKα (Thr23) and p65 activation increase during skin aging

**Figure 2:**
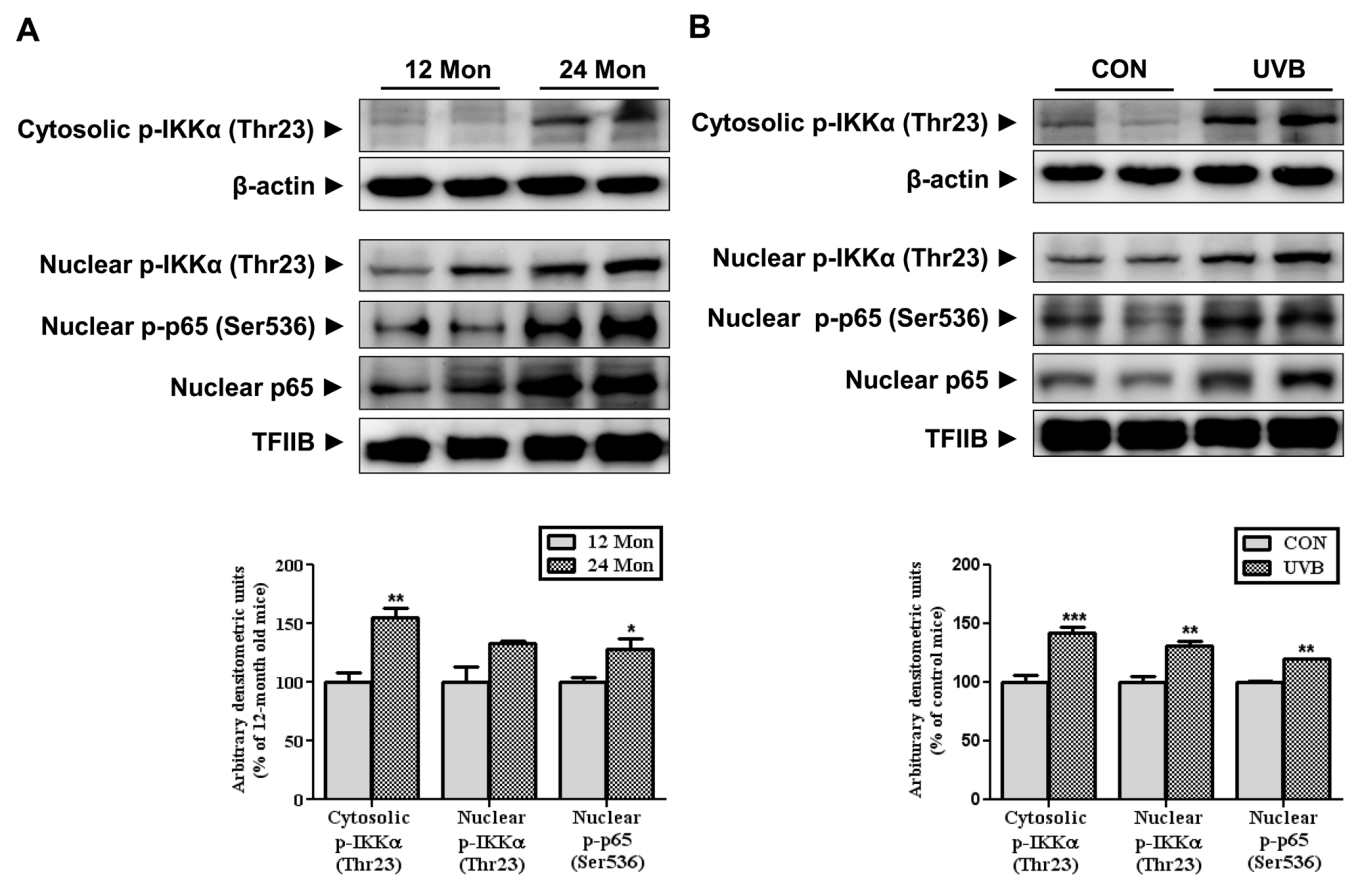
The levels of IKKα and p65 phosphorylation during skin aging Western blotting was performed to detect the levels of IKKα phosphorylated at Thr23 and p65 at Ser536 in skin homogenates from 12- and 24-month-old mice (**A**) and in skin homogenates of control and UVB-irradiated mice (**B**) Blots were quantified by densitometry and normalized to β-actin or TFIIB. Bars represent the mean percentage value ± SEM in 12-month-old mice (*n* = 7-8, * *p* < 0.05, ** *p* < 0.01 *vs*. 12-month-old mice) and control mice (*n* = 7-8, * *p* < 0.05, ** *p* < 0.01, *** *p* < 0.001 *vs*. control mice).

We investigated the downstream targets of mTORC2/Akt which are involved in skin aging. Previous study has reported that Akt-mediated phosphorylation of IKKα at Thr23 induces its translocation to the nucleus, leading to the activation of NF-κB [9]. Nuclear IKKα induces the post-translational modification of the p65 subunit of NF-κB at Ser536, enhancing NF-κB activity through increasing its DNA binding activity [11]. Therefore, we measured the level of IKKα phosphorylated at Thr23 in both models of skin aging. Interestingly, the phosphorylation of IKKα was increased in the cytosolic and nuclear fractions by physical age (Figure 2A) and UVB irradiation (Figure 2B) which results were consistent with mTORC2 activation. Also, nuclear p65 and its phosphorylation at Ser536 was upregulated, which is known to cause its transactivation during skin aging (Figure 2).

### Upregulation of the mTORC2/Akt/IKK signaling pathway and NF-κB activation by UVB irradiation in HaCaT cells

**Figure 3:**
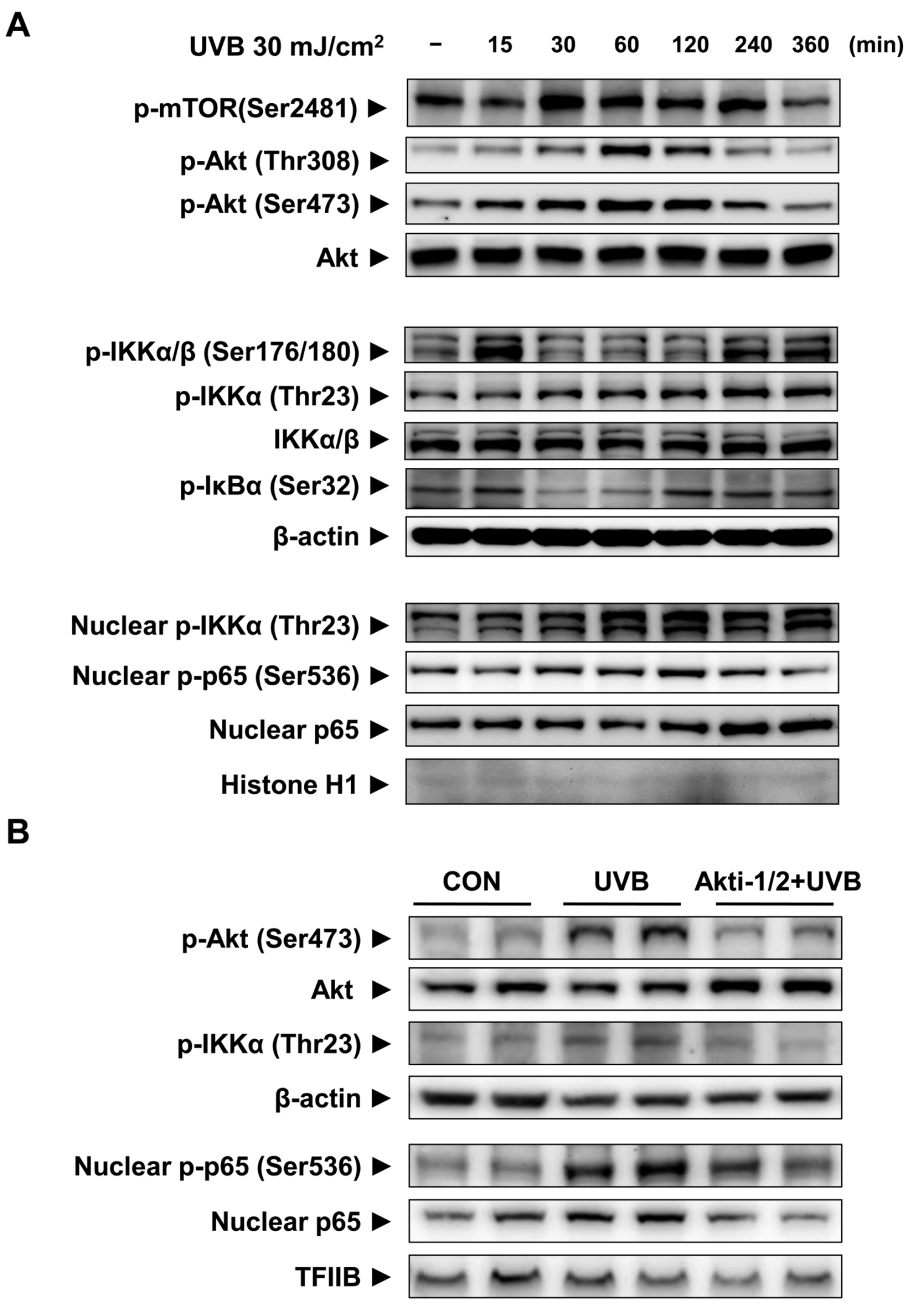
The activation of mTORC2 and its downstream signaling by UVB irradiation in HaCaT cells HaCaT cells were incubated for 15 min - 6 h after UVB irradiation (30 mJ/cm^2^) (**A**) Western blotting was performed to detect mTORC2, activation of the Akt signaling pathway, as indicated by the presence of phosphorylated Akt and changes in the IKKαβ/IκBα signaling pathway in the cytosolic fraction. Nuclear phosphor-IKKα/β (Thr23), phosphor-p65 (Ser536), and total p65 were measured by Western blotting using specific antibodies. To clarify whether Akt regulates IKKα activity, HaCaT cells were treated with 1 μM of the Akt inhibitor Akti-1/2, 1 h prior to UVB irradiation (60 mJ/cm^2^) (**B**). Two hours after UVB irradiation Western blotting was performed to detect changes in the signaling pathways and the activation of p65 in the cytosolic and nuclear fractions. β-Actin, histone H1, and TFIIB blots were shown to clarify the same amount of protein loaded in cytosolic and nuclear fractions. For each protein, one representative blot is shown from 3 experiments that yielded similar results.

To confirm *in vivo* results, we irradiated HaCaT cells with UVB and monitored mTORC2 activity and its downstream signaling in the time-dependent manner. The phosphorylations of mTORC2 and Akt at Ser473 was time-dependently induced and quenched 6 h after UVB exposure (Figure 3A). Phospho-IKKαβ (Ser176/180) which was phosphorylated by other upstream kinases like NF-κB-inducing kinase (NIK) and transforming growth factor-β (TGFβ)-activated kinase-1 (TAK1) [12] were elevated both early and late in the time course. UVB irradiation stimulated Akt-mediated phosphorylation of IKKα at Thr23 in both the cytoplasm and nucleus (Figure 3A). Similar to the change in nuclear phospho-IKKα (Thr23) levels, phospho-p65 (Ser536) was elevated (Figure 3A). We detected the phosphorylation of inhibitor of NF-κB α (IκBα) at the low level of phosphorylated IKKα/β at Ser176/180, which showed phosphorylated form of IKKα at Thr23 can induce the activation of NF-κB.

To further examine Akt-dependent activation of NF-κB by UVB irradiation, Akt activity was blocked by its small inhibitor Akti-1/2. Suppression of Akt activity reduced UVB-induced phosphorylation of IKKα at Thr23 and the nuclear presence and phosphorylation of p65 (Figure 3B). These date clearly showed that Akt is implicated in the activation of NF-κB through IKKα activation.

### Regulatory role of mTORC2 in NF-κB activation by UVB irradiation through Akt/IKKα

**Figure 4:**
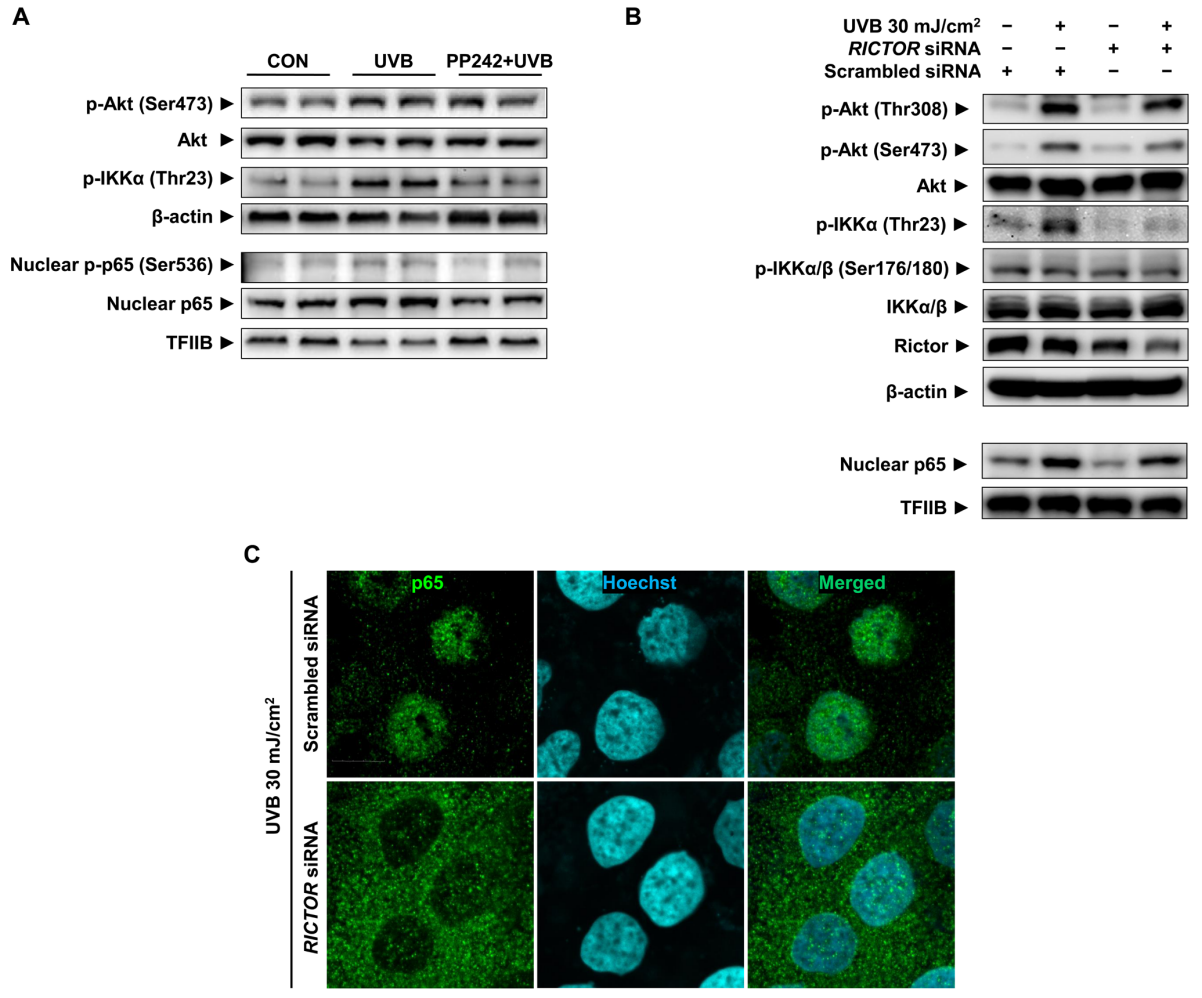
Attenuation of UVB-induced p65 activation by mTORC2 inhibition in HaCaT cells HaCaT cells were treated with pp242 (**A**) or transfected with *RICTOR* siRNA (**B**) and (**C**) to inhibit mTORC2 activity and then irradiated by 30 mJ/cm^2^ of UVB. Scrambled siRNA was used as a negative control. The levels of Rictor, phosphor-Akt (Ser473 and Thr308), phospho-IKKs, and nuclear p65 were measured by Western blotting (A and B) and confocal microscopic analysis detected p65 translocation (C). One representative experiment is shown from 3 experiments that yielded similar results.

To establish a causal relationship between mTORC2 and activation of NF-κB by UVB, we applied pp242, a specific inhibitor of mTOR, and *RICTOR* siRNA. First, UVB-induced IKKα/NF-κB activation was downregulated by the pre-treatment of mTOR kinase inhibitor, pp242 (Figure 4A). Furthermore, we verified the specific role of mTORC2 in the activation of NF-κB using *RICTOR* siRNA to suppress only mTORC2 activity, because pp242 can inhibit the functions of both mTORC1 and mTORC2. The stability and integrity of mTORC2 is dependent on the presence of the core subunits RICTOR and SIN1. The knockdown of RICTOR reduces mTOR-RICTOR interaction, resulting in impaired mTORC2 formation and activity [13, 14]. UVB-induced phosphorylation of Akt was reduced in cells transfected with *RICTOR* siRNA compared to scrambled siRNA-transfected cells, implying a reduction of mTORC2 activity. The reduced activity of mTORC2 attenuated IKKα phosphorylation at Thr23 but did not affect the phosphorylation of IKKα/β at Ser176/180. Correspondingly, the attenuated mTORC2 activity partially inhibited UVB-induced nuclear localization and phosphorylation of p65 in *RICTOR*-knockdown HaCaT cells (Figure 4B). The confocal microscopy also revealed that UVB-induced translocation of p65 to nucleus was suppressed in *RICTOR*-knockdown cells (Figure 4C).

## DISCUSSION

**Figure 5:**
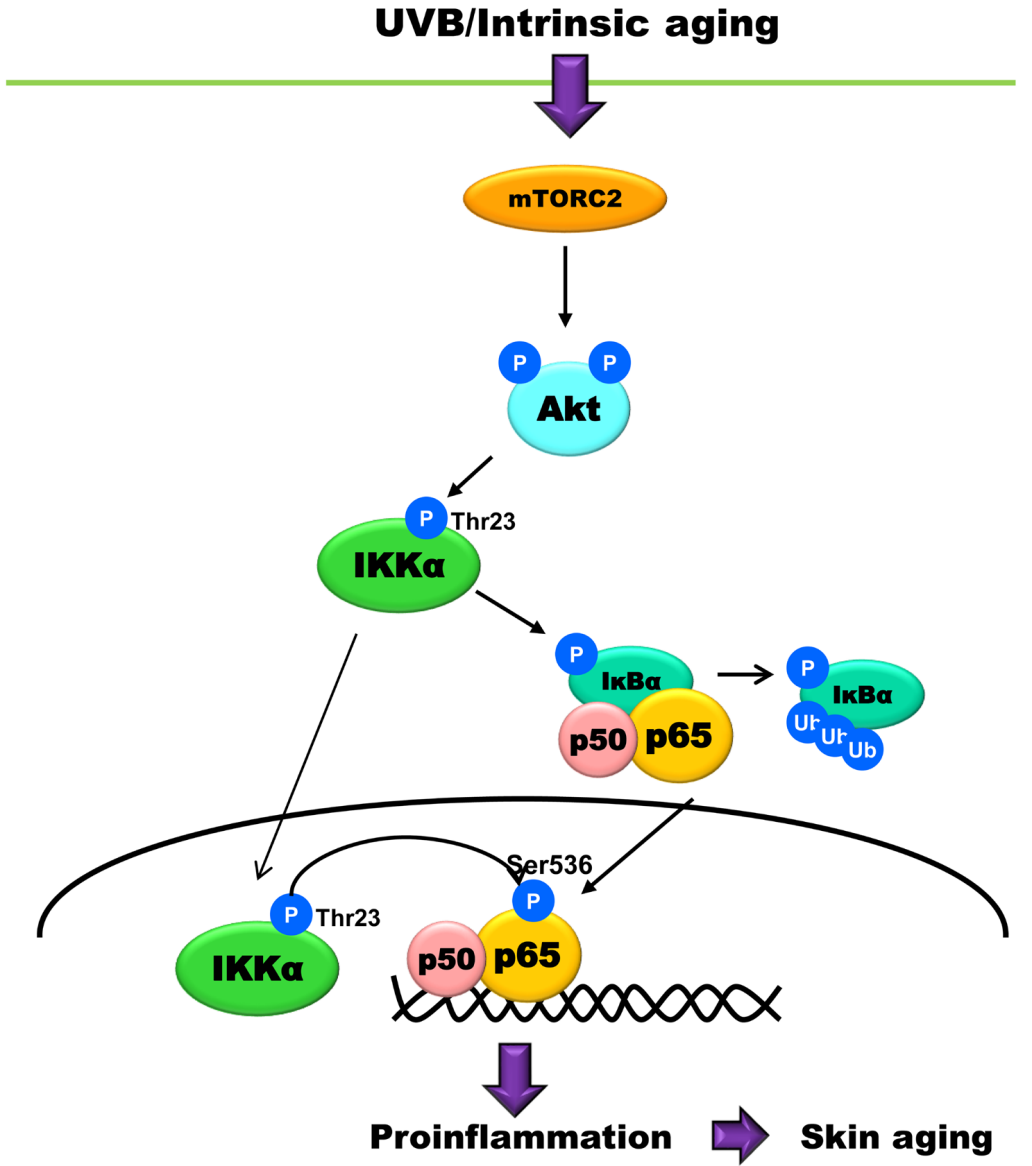
A schematic model showing the underlying mechanism of skin aging UVB or age increases the activity of mTORC2 that phosphorylates Akt. In turn, Akt phosphorylates IKKα at Thr23, leading to the translocation and phosphorylation of p65 during skin aging.

The current study demonstrates the upregulation of mTORC2 signaling pathway and its role in skin aging. So far studies focusing on mTOR in the skin were limited to skin cancer and the role of mTORC1. mTORC2, a major regulator of Akt, has the potential to modulate various substrates of Akt such as Forkhead box protein O 1 and 3, a well-known substrate of Akt [15]. Our data clearly showed that mTORC2 significantly upregulated during intrinsic and UVB-induced skin aging. Also, the inhibition of mTORC2 activity suppressed UVB-induced activation of NF-κB in the Akt-dependent manner, indicating that NF-κB is one of downstream targets regulated by mTORC2 (Figure 5).

It has been reported that age-related alteration of mTORC2 activity in the other tissues. The recent paper authored by Baar et al showed the increased mTORC2 signaling in liver, muscle, and heart, but not adipose tissue [16] and we also confirmed the phosphorylated mTOR at ser2481 was increased in the aged liver (Supplementary Figure 1). mTORC2 could be considered as the mechanism underlies aging of other tissues as well as skin. Skin undergoes not only intrinsic aging but also extrinsic factors-induced aging including photoaging due to its direct contact with harsh external environmental conditions [17]. Here, our evidence revealed the implication of mTORC2 signaling pathway in both intrinsic aging and photoaging, which is beneficial to be good target to achieve skin aging retardation.

Mounting evidence shows that NF-κB is one of the most vulnerable targets that is activated by both UVB and age [18-21]. NF-κB is activated during photoaging or upon UV irradiation, and induces various proinflammatory genes. Previously, our group reported that NF-κB activation leads to the gene expression of several pro inflammatory cytokines and plays a central role during aging [22]. Genetic inhibition of NF-κB in the skin of aged mice restores the global gene expression profiles and tissue characteristics to that of young mice, demonstrating NF-κB as an important mediator of aging [23].

A striking finding obtained in this study is that NF-κB is regulated through the Akt/IKKα signaling pathway during skin aging. There are several signaling pathways capable of activating NF-κB. Photoaging and UV irradiation are known to activate MAPK signaling pathways, JNK, ERK, p38, and the Akt signaling pathway [24]. In the intrinsically aged skin of mice, we observed the distinct activation of Akt. While the changes in MAPKs signaling could not provide the association with NF-κB activation during intrinsic skin aging (data not shown). Only JNK activation was detected, which is not responsible for NF-κB activation. The role of Akt in the regulation of NF-κB activation has not been thoroughly explored during aging, but the upregulations of Akt and NF-κB in response to UV irradiation is well documented. The precise interaction between Akt and NF-κB in photoaging was not well understood until now. Here, we showed that Akt-dependent IKKα phosphorylation led to the activation of NF-κB in response to UVB. Consistent with this finding, IKKα phosphorylation at Thr23 was significantly elevated in both intrinsic and UVB-irradiated skin *in vivo*.

In conclusion, this study showed the upregulation of mTORC2 activity and Akt/IKKα signaling pathway during skin aging and examines the effect of this upregulation on the activation of NF-κB (Figure 5). We propose that the mTORC2/Akt/IKKα/NF-κB signaling pathway is one important pathway that is responsible for both intrinsic and UVB-induced skin aging.

## MATERIALS AND METHODS

### Animals

SPF male C57BL/6 mice at 12 and 24 months of age were obtained from Samtako (Osan, Korea) (*n* = 7-8). After a 1-week stabilization period, the mice were sacrificed, their dorsal skins were saved with a razor, and were immediately collected and frozen in liquid nitrogen and stored at -80^o^C. SPF 6-week-old male Hos:HRM-2 melanin-possessing hairless mice were obtained from the Hoshino Laboratory Animals (Saitama, Japan). After a 1-week stabilization period, the mice were randomly divided into 2 groups of 8 animals each and were exposed to 150 mJ/cm^2^ UVB every other day for 16 days and then every day for 3 days before sacrifice (Table 1) [25]. The UVB light source was an UV crosslinker (UVP, Upland, CA) with 302 nm peak emission (wavelength range, 290-340 nm). The animal protocol used in this study was reviewed and approved by the Pusan National University-Institutional Animal Care and Use Committee (PNU-IACUC).

**Table 1:** Experimental schedule of UVB treatment

Day	1	2	3	4	5	6	7	8	9	10	11	12	13	14	15	16	17	18	19
UVB 150 (mJ/cm^2^)	x		x		x		x		x		x		x		x		x	x	x

Note: x indicates a single treatment with UVB.

### Cells

HaCaT cells, a human keratinocyte cell line, were obtained from the American Type Culture Collection (Manassas, VA). Cells were grown in Dulbecco's modified eagle medium (HyClone Laboratories, Utah) containing 2 mM l-glutamine, 100 units/ml penicillin, 100 μg/ml streptomycin, and 10% heat-inactivated fetal bovine serum (HyClone). Cells were maintained at 37^o^C in a humidified 5% CO_2_ atmosphere. Media were replaced by Dulbecco's phosphate-buffered saline (D-PBS, GIBCO^®^ Grand Island, NY) prior to irradiation of UVB. After exposure to UVB, the cells were incubated in serum-free medium (SFM). Akt1/2 kinase inhibitor (Akti-1/2, Sigma, Saint Louis, MO) and pp242 (Sigma) were dissolved into DMSO and used at 1 μM and 0.1 μM, respectively.

### Protein isolation and cell fractionation

Cell fractionation was performed to separate cytosolic and nucleic fraction as previously described (14). Briefly, skin tissue or cells were homogenized in hypotonic lysis buffer and added with 10% NP-40 solution, and centrifuged at 14,000 × g for 2 min. Supernatants were deemed the cytosolic fractions. Pelleted nuclei were washed twice with hypotonic lysis buffer and suspended in nuclear extraction buffer, kept on ice for 30 min, and centrifuged at 14,000 g for 10 min. Supernatants were collected as nuclear proteins. TFIIB and β-actin were used for the validation of fractionation (Supplementary Figure 2). Whole cell lysates were prepared using RIPA.

### Western blot analysis

Western blot analyses were carried out as described previously (14) using the antibodies detailed below. Antibodies against p-mTOR (Ser2481), Rictor, p-Akt (Thr308), p-Akt (Ser473), Akt, and p-IKKα/β (Ser176/180), were purchased from Cell Signaling Technology (Beverly, MA). Antibodies against p-IKKα (Thr23), IKKα/β, p-IκB (Ser32), p65, p-p65 (Ser536), TFIIB, Histone H1, and β-actin were obtained from Santa Cruz Biotechnology (Santa Cruz, CA).

### RNA interference

Rictor-specific small-interfering RNA were purchased from Integrated DNA Technologies (Coralville, Iowa). The Rictor siRNA sequences were as follows: 5′-CUA GCU UUC UCA UAU UUG AUA CUC CCU-3′. siRNAs were transfected using Lipofectamine 2000 (Invitrogen, Carlsbad, CA) according to the manufacturer's instructions. One day prior to transfection, cells were seeded in 6-well plates or 60 mm dishes in appropriate growth medium (without antibiotics) and allowed to attach for 24 h. Cells were 50-60% confluent at the time of transfection. The final concentration of siRNA was 25 nM. Cells were incubated at 37^o^C for 48 h prior to the gene knock-down assay.

### Immunofluorescence

Cell were seeded in a covered glass-bottom-dish (SPL Labware, Seoul, Korea) and transfected with siRNA as described above. Two hour after UVB irradiation cells processed for immunofluorescence. Cells were fixed in 4% paraformaldehyde for 30 min and washed with PBS. After rinsing in PBS with 0.5% Triton-x 100 (PBST), cells were blocked in ABS/0.1% Triton X-100/3% goat serum (ABS-TS) at room temperature for 30 min, and incubated with p65 antibody (Santa Cruz) diluted in ABS-TS at 4^o^C overnight. After washing with ABS-TS, cell were incubated with AlexaFluor-conjugated secondary antibodies (Invitrogen) and stained with Hoechst 33342 (Invitrogen). Confocal images were obtained using an FV10i FLUOVIEW Confocal Microscope (Olympus, Tokyo, Japan) and processed with Photoshop 5.5 CS.

### Statistical analysis

One-way analysis of variance (ANOVA) was used to evaluate the differences between 3 or more groups. Differences between the mean of individual groups were assessed using Bonferroni's post hoc test. The Student's t-test was used to analyze the difference between 2 groups. *P* values <0.05 were considered statistically significant. Analyses were performed using GraphPad Prism 5 (GraphPad Software, La Jolla, CA).

## SUPPLEMENTARY MATERIAL FIGURES


