# Supplementary material for: The underlying mechanism of proinflammatory NF-κB activation by the mTORC2/Akt/IKKα pathway during skin aging
Source: Oncotarget. 2016 Jul 29;7(33):52685–94. doi: 10.18632/oncotarget.10943 (PMC5288141; doi:10.18632/oncotarget.10943)
Supplement: Supplementary file 1 [file oncotarget-07-52685-s001.pdf]

The underlying mechanism of proinflammatory NF-κB activation by the mTORC2/Akt/IKKα pathway during skin aging

Supplementary Material

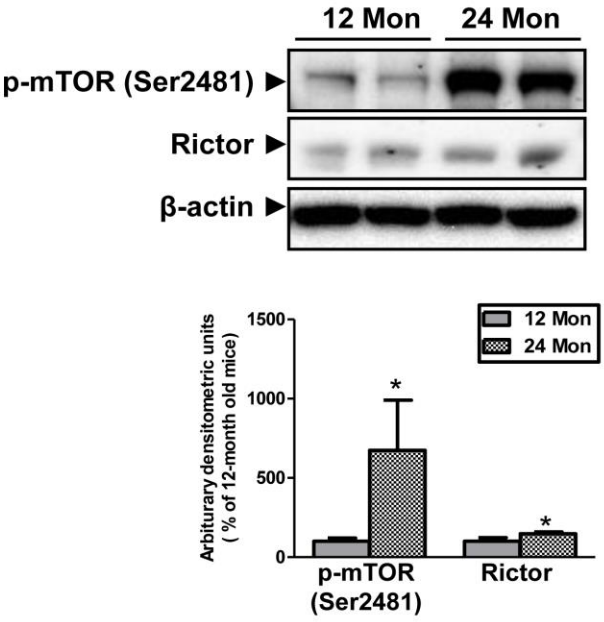

Supplementary figure 1. Alteration of mTORC2 activity in aged liver. Western blotting analysis of the level of mTOR phosphorylated at Ser2481 and Rictor in liver homogenates of 12- and 24-month-old mice The blots were quantified by densitometry. The blots of phospho-mTOR (Ser2481) and Rictor were normalized to β-actin. Bars represent the mean percentage value ± SEM in 12-month-old mice (n = 6, *p < 0.05 vs. 12-month-old mice).

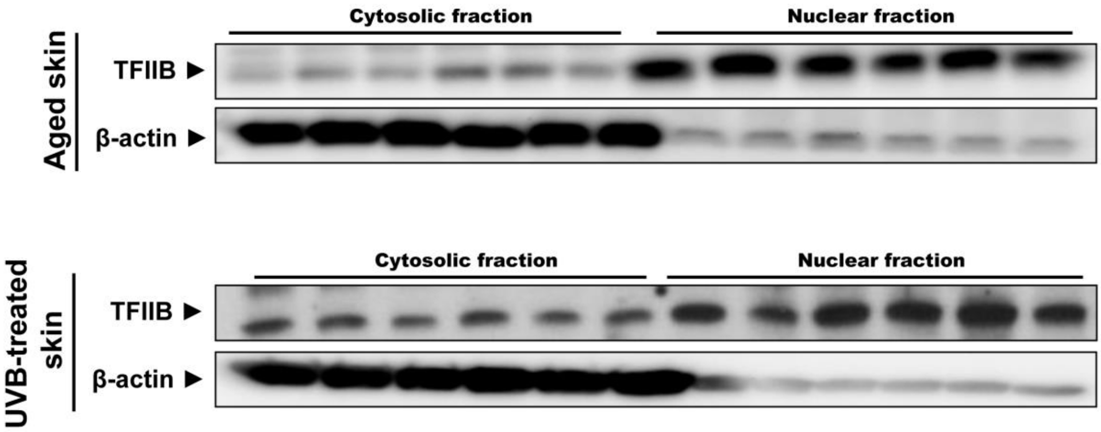

Supplementary figure 2. Validation of fractionation of skin homogenate. Western blotting analysis of TFIIB and β-actin to verify the fractionation of skin homogenates. Three samples were randomly chosen from each group.
